# Reverse transcription quantitative real-time polymerase chain reaction reference genes in the spared nerve injury model of neuropathic pain: validation and literature search

**DOI:** 10.1186/1756-0500-6-266

**Published:** 2013-07-10

**Authors:** Nicolas Piller, Isabelle Decosterd, Marc R Suter

**Affiliations:** 1 Pain Center, Department of Anesthesiology, University Hospital Center and University of Lausanne, Avenue du Bugnon 46, 1011 Lausanne, Switzerland; 2 Department of Fundamental Neurosciences, University of Lausanne, Lausanne, Switzerland

**Keywords:** Neuropathic pain, Spared nerve injury, Reference gene, RT-qPCR, Rat

## Abstract

**Background:**

The reverse transcription quantitative real-time polymerase chain reaction (RT-qPCR) is a widely used, highly sensitive laboratory technique to rapidly and easily detect, identify and quantify gene expression. Reliable RT-qPCR data necessitates accurate normalization with validated control genes (reference genes) whose expression is constant in all studied conditions. This stability has to be demonstrated.

We performed a literature search for studies using quantitative or semi-quantitative PCR in the rat spared nerve injury (SNI) model of neuropathic pain to verify whether any reference genes had previously been validated. We then analyzed the stability over time of 7 commonly used reference genes in the nervous system – specifically in the spinal cord dorsal horn and the dorsal root ganglion (DRG). These were: Actin beta (Actb), Glyceraldehyde-3-phosphate dehydrogenase (GAPDH), ribosomal proteins 18S (18S), L13a (RPL13a) and L29 (RPL29), hypoxanthine phosphoribosyltransferase 1 (HPRT1) and hydroxymethylbilane synthase (HMBS). We compared the candidate genes and established a stability ranking using the geNorm algorithm. Finally, we assessed the number of reference genes necessary for accurate normalization in this neuropathic pain model.

**Results:**

We found GAPDH, HMBS, Actb, HPRT1 and 18S cited as reference genes in literature on studies using the SNI model. Only HPRT1 and 18S had been once previously demonstrated as stable in RT-qPCR arrays. All the genes tested in this study, using the geNorm algorithm, presented gene stability values (M-value) acceptable enough for them to qualify as potential reference genes in both DRG and spinal cord. Using the coefficient of variation, 18S failed the 50% cut-off with a value of 61% in the DRG. The two most stable genes in the dorsal horn were RPL29 and RPL13a; in the DRG they were HPRT1 and Actb. Using a 0.15 cut-off for pairwise variations we found that any pair of stable reference gene was sufficient for the normalization process.

**Conclusions:**

In the rat SNI model, we validated and ranked Actb, RPL29, RPL13a, HMBS, GAPDH, HPRT1 and 18S as good reference genes in the spinal cord. In the DRG, 18S did not fulfill stability criteria. The combination of any two stable reference genes was sufficient to provide an accurate normalization.

## Background

While 20%-30% of the general populace are affected by chronic pain, a significant proportion, about 7%, experiences neuropathic pain characteristics [1,2]. Defined by the International Association for the Study of Pain (IASP) as a pain “caused by a lesion or disease of the somatosensory nervous system”, neuropathic pain represents a daily challenge to medical practice and a worldwide public health problem since it is often refractory to treatment. Several animal models of neuropathic pain are used to study its different pathophysiological mechanisms and hopefully uncover new targets for treatment. Among them, the spared nerve injury (SNI) model [3] represents an easy-to-perform and robust model of peripheral nerve injury. The axotomy of the tibial and peroneal branches of the sciatic nerve, sparing the sural nerve, allows a straightforward testing of the hind paw’s sural nerve territory for signs of hyperalgesia. After a peripheral nerve injury in rodents, numerous alterations in the somatosensory nervous system can be observed, which together lead to a hyperexcitability of the system [4]. Besides changes affecting the primary nociceptors whose cell bodies are in the dorsal root ganglion (DRG) or the secondary neurons in the dorsal horn projecting into the brain, glial cells also react dramatically to a peripheral nerve injury like SNI. A common approach used to elucidate such changes in molecular machinery is to explore modifications in the expression of relevant genes [5,6]*.* These variations can be detected and quantified in a sensitive, specific way using a reverse transcription quantitative real-time polymerase chain reaction (RT-qPCR), assuming that an accurate normalization has been performed with reference genes that have proved stable in all biological replicates and experimental conditions [7-10]. The recently published guidelines on the “minimum information for publication of quantitative real-time PCR experiments” (MIQE) strongly recommended the validation of reference genes used in different cell types and conditions [7]. These guidelines outline the importance of RT-qPCR accuracy and recommend following detailed procedures to produce the most robust and reproducible RT-qPCR data.

Algorithms like geNorm or NormFinder were developed to help researchers assess candidate reference genes. GeNorm has been well described [9] and widely accepted as a very useful tool for the normalization of RT-qPCR data [11]. It measures the stability of potential reference genes by comparing their expression levels against one another. The expression ratio is supposed to be the same in all biological and technical replicates. This pairwise comparison permits the assessment and ranking of candidate reference genes and additionally demonstrates how many are necessary for an accurate normalization [9,11]. To our knowledge, a validation of reference genes for the rat after spared nerve injury had never been published. We therefore performed a literature search for all studies using quantitative or semi-quantitative PCR in the SNI model and listed all the reference genes used. We then tested a total of 7 genes – including commonly used reference genes in the nervous system and those used in the SNI model – at different time points after nerve lesion in the spinal cord dorsal horn and in the DRG. These genes were Actin beta (Actb), Glyceraldehyde-3-phosphate dehydrogenase (GAPDH), ribosomal proteins 18S (18S), L13a (RPL13a) and L29 (RPL29), hypoxanthine phosphoribosyltransferase 1 (HPRT1) and hydroxymethylbilane synthase (HMBS) [12-15]. Selected genes were related to various metabolic pathways to avoid any co-regulation [9].

## Methods

### Animals

We carried out our experiments on Sprague–Dawley rats (Charles River, L’Abresle, France) weighing 250–300 grams. They were housed under 12 h day/night photoperiodic conditions, at a constant temperature and with free access to water and food. All procedures were approved by the Canton of Vaud’s Animal Experimentation Committee and were in accordance with the Swiss Federal Law on Animal Welfare and the IASP’s guidelines [16].

### Surgery

Rats were anesthetized with isoflurane 1.5%-2.5% (Abott, Baar, Switzerland) and surgical procedures were performed as previously described [3,17]. Briefly, the left sciatic nerve was exposed at the mid-thigh level distal to the trifurcation, tibial and common peroneal branches were tightly ligated with 5.0 silk and axotomized, leaving the sural branch intact. Muscle and skin were closed in two layers and animals were allowed to recover. Three rats were sacrificed at 2, 4, 7, 10, 14 and 21 days after surgery.

### Literature search

We performed a literature search in the PubMed database using the keywords “spared nerve injury” and selected every study using semi-quantitative PCR or RT-qPCR in the rat SNI model (13 April 2013). We only selected articles in English. Reference genes used were noted, as were indications of their validation in the models.

### RNA isolation and reverse transcription

Animals were transcardially perfused with NaCl 0.9% in order to expel the blood from collected tissue. Ipsilateral L4 and L5 DRG and spinal cord dorsal horn were rapidly dissected and immediately stored in RNAlater® (Qiagen, Switzerland) for stabilization at 4°C overnight, and then frozen at −80°C until processed. Samples were first homogenized with a POLYTRON® homogenizer and then total RNA was extracted using the RNeasy Plus Mini Kit (Qiagen). To ensure complete removal of gDNA, we treated the dorsal horn samples with a DNase (RNAqueous®-4PCR Kit, Ambion) and then the inactivation reagent recommended in the manufacturer’s instructions.

Nucleic acid purity was assessed measuring the A_260_/A_280_ ratio by spectrophotometer (NanoDrop). Total RNA integrity (RIN) and quantity were determined with an Agilent 2100 Bioanalyser (Agilent Technologies). RNA used had to have both a A_260_/A_280_ ratio between 1.8 and 2.1, as well as a RIN ≥8 for dorsal horn RNA extractions and ≥6.9 for DRG samples. The reverse transcription was achieved using Omniscript RT Kit (Qiagen), according to manufacturer’s instructions. All reactions took place for 1h at 37°C in a final volume of 40 μl containing 1 μg total RNA in the presence of 20 units RNase inhibitor (RNasin® Ribonuclease Inhibitor, Promega), 1 μg random hexamers (Microsynth), 4 μl 10× Buffer RT, 0.5 mM dNTPs and 8 units Omniscript reverse transcriptase (Omniscript RT Kit, Qiagen).

### Selection of reference genes and primer design

Reference genes were chosen from those used previously in the SNI model. Other potentially suitable reference genes were selected among those used in published literature on the nervous system (Table 1). Whenever possible, primers fulfilled the following recommended criteria: amplicon length of 60 bp-150 bp, location of primers on two different exons, primer sequence length of 18 bp-25 bp, melting temperature of 60°C +/−1°C and GC content of 40%-60% [7,18-20]. Primer specificity was checked *in silico* (Primer-BLAST Tool from http://www.ncbi.nlm.nih.gov/tools/primer-blast/) [21]. All oligonucleotides were supplied unmodified and desalted (Microsynth AG, Switzerland).

**Table 1:** Specification of used reference genes

**Gene symbol**	**Sequence (5'->3')**	**Position**	**Product length**	**Intron inclusion**	**RTqPCR efficiency in DH/DRG**	**Ref.**
**Full name**						
**Accession number**						
**18S**	Fw: GGCTCATTAAATCAGTTATGGTTCCT	94-119	147	no	78%/70%	[22]
18S ribosomal RNA	Rev: GTTGGTTTTGATCTGATAAATGCACG	240-215				
V01270						
**RPL29**	Fw: ACAGAAATGGCATCAAGAAACCC	96-118	105	yes	81%/77%	[15]
Ribosomal protein L29	Rev: TCTTGTTGTGCTTCTTGGCAAA	200-179				
NM_017150						
**RPL13a**	Fw: TCTCCGAAAGCGGATGAACAC	185-205	145	yes	88%/73%	[14]
Ribosomal protein L13A	Rev: CAACACCTTGAGGCGTTCCA	329-310				
NM_173340						
**Actb**	Fw: GGAGATTACTGCCCTGGCTCCTA	1023-1045	150	yes	85%/63%	[14]
Actin, beta	Rev: GACTCATCGTACTCCTGCTTGCTG	1172-1149				
NM_031144						
**HPRT1**	Fw: GCATCTAAGAGGTTTCCCCAGT	1133-1154	76	no	70%/74%	^1^
Hypoxanthine phosphoribosyl-transferase 1	Rev: GCATTTAAAAGGAACGGTTGAC	1208-1187				
NM_012583						
**HMBS**	Fw: GAGACCATGCAGGCCACCAT	1007-1026	97	yes	78%/72%	[15]
Hydroxymethyl-bilane synthase	Rev: TTGGAATGTTCCGGGCAGTG	1084-1103				
NM_013168						
**GAPDH**	Fw: CCCCCAATGTATCCGTTGTG	780-799	118	yes	89%/87%	[23]
Glyceraldehyde-3-phosphate	Rev: TAGCCCAGGATGCCCTTTAGT	877-897				
dehydrogenase						
NM_017008						

*Fw* forward, *Rev* reverse primer sequence, *DH* dorsal horn, *DRG* dorsal root ganglion.

^1^ from our laboratory database.

### Quantitative real-time PCR (qPCR)

We performed qPCR on an iQ5 Cycler (Bio-Rad) with SYBR Green I. The reactions were carried out in 96-well plates (Thermo-Fast® 96 Semi-Skirted PCR Plate, Thermo Scientific, Switzerland) each with a total volume of 20 μl. Each well contained 5 μl of a 100-fold dilution of cDNA, 10 μl of iQ™ Sybr® Green Supermix (2× qPCR mix contains dNTPs, 50 U/ml iTaq DNA polymerase, 6 mM MgCl_2_, SYBR Green I, enhancers, stabilizers, 20 nM fluorescein) (Bio-Rad, Switzerland), 2 μl of each primer 1–3 μM and 1 μl water. We optimized qPCR conditions on the iQ5 thermal gradient cycler and by testing different concentrations of primers and templates. The qPCR program began with an initial three-minute denaturation step at 95°C to activate the hot-start iTaq™ DNA polymerase. This was followed by 45 cycles of 10 s at 95°C for denaturation and 45 s at 60°C for annealing and extension.

We confirmed the amplification of specific qPCR products by performing a melting-curve step at the end of each run. Serial dilution curves for each primer allowed us to calculate qPCR efficiencies. The 100-fold diluted cDNA utilized for all the amplifications was within the linear dynamic range of the calibration curve – between 10 and 1000-fold dilution. Across all the assays, none of the quantification cycle (Cq) values was higher than 30. No-template and no-reverse transcription controls were run to determine any contamination or the generation of primer dimers. All amplifications were run in triplicate, and any doubtful curves were excluded. To minimize technical variation between samples through different runs we preferred the sample maximization method [11], i.e. a run contained all the samples for one gene of interest respective to one reference gene.

### Data analysis

Raw data was collected and computed by iQ5 BioRad software with an automated analysis of the baseline and threshold of each run, and then exported in Excel files for further analyses. We rescaled all Cq values for each gene to the lowest Cq value as an internal control, converted these rescaled Cq logarithmically into linear, relative quantities taking into account the gene specific amplification efficiency [relative quantity = (1+efficiency) ^ (Cq^internal control^-Cq^sample^)]. Finally, we calculated arithmetical means from the replicates [11,24]. To explore the stability of each candidate gene we calculated M-values from the geNorm algorithm and coefficients of variation (CV) [9,11]. The M-value corresponds to the average pairwise variation between a gene and the other candidate genes. The more this value tends to zero, the more stably the gene is expressed in comparison to the others. For a heterogeneous sample, a candidate gene with an M-value below 1 can be considered as a reliable reference gene [11]. The CV for a gene was obtained by calculating the ratio of the standard deviation (σ) to the mean (μ) of relative quantities (CV = σ / μ). For a heterogeneous panel, a value below 50% is proposed as satisfactory [11].

To rank the genes that satisfied the above criteria, M-values were recalculated after stepwise elimination of the worst candidate. To assess the number of reference genes needed for an accurate normalization, the effect of inclusion of a supplementary gene to those already considered stable was determined by calculating the pairwise variation of the added candidate gene to the others (standard deviation of logarithmically transformed expression ratios). If this pairwise variation goes below 0.15, no supplementary gene is needed [9].

## Results

### Reference genes used in the SNI model

We found 484 articles in the PubMed database using “spared nerve injury” as keywords. Twenty-six articles were retrieved with semi-quantitative PCR or RT-qPCR performed in the rat DRG, spinal cord or brain. Despite its rigorous validation never having been mentioned, GAPDH was the most commonly used reference gene (16 times) whether in the DRG or in the dorsal horn. This was followed by Actb and 18S (3 times), HPRT1 (twice) and HMBS (once). HPRT1 and 18S were the only two reference genes with validation in the rat SNI model (Table 2).

**Table 2:** List of reference genes in the rat spared nerve injury model

**Article**	**PCR method**	**Sample**	**Reference genes**	**Validation**
Kanda et al., 2013 [25]	PCR	L4/L5 SC	GAPDH	no
Zhou et al., 2013 [26]	RT-qPCR	L4/L5 DRG	GAPDH	no
Kashimoto et al., 2013 [27]	RT-qPCR	L4/L5 SC	GAPDH	no
Shankarappa et al., 2012 [28]	RT^2^ Profiler PCR array	L4/L5 DRG	HPRT1	validated^1^
Inquimbert et al., 2012 [29]	PCR	L4/L5 DH	GAPDH	no
Zapata et al., 2012 [30]	RT-qPCR	RVM	18S and HMBS	no
Samad et al., 2013 [31]	PCR	L4 DRG	GAPDH	no
Kobayashi et al., 2012 [32]	RT-qPCR	L4/L5 SC	GAPDH	no
Liu et al., 2012 [33]	RT-qPCR	L4–L6 SC	Actb	no
Tochiki et al., 2012 [34]	PCR	L4-L6 DH	HPRT1	no
Okubo et al., 2012 [35]	RT-qPCR	L4-L5 SC	GAPDH	no
Del Rey et al., 2011 [36]	PCR	hippocampus	not mentioned	no
Kühlein et al., 2011 [37]	RT-qPCR and PCR	DH and DRG	Actb and 18S	no
Yamanaka et al., 2011 [38]	RT-qPCR	L4, 5 DRG	GAPDH	no
de Novellis et al., 2011 [39]	RT-qPCR	mPFC	Actb	no
Vega-Avelaira et al., 2009 [40]	PCR	L4/L5 DRG	GAPDH	no
Costigan et al., 2009 [41]	PCR	L4/L5 DH	GAPDH	no
Okubo et al., 2010 [42]	RT-qPCR	L4-L5 SC	GAPDH	no
Staaf et al., 2009 [43]	Taqman Low Density Arrays	L4 DRG	18S	validated^2^
Moss et al., 2008 [44]	PCR	L4/5 DRG	GAPDH	no
Berta et al., 2008 [45]	PCR	L4 and L5 DRGs	GAPDH	no
Millecamps et al., 2007 [46]	PCR	mPFC	GAPDH	no
Moss et al., 2007 [47]	PCR	L4/L5 dorsal horn	GAPDH	no
Apkarian et al., 2006 [48]	RT-qPCR	brainstem, thalamus, and prefrontal cortex	GAPDH	no
Pertin et al., 2005 [49]	PCR	L4/L5 DRG	GAPDH	no
Takahashi et al., 2003 [50]	RT-qPCR	L5 DRG	GAPDH	no

Literature search in the PubMed database of studies using semi-quantitative PCR or RT-qPCR in the rat SNI model.

*PCR* semi-quantitative polymerase chain reaction, *RT-qPCR* reverse transcription quantitative real-time polymerase chain reaction, *SC* spinal cord, *DRG* dorsal root ganglion, *DH* dorsal horn, *RVM* rostral ventromedial medulla, *PL-IL* prelimbic and infralimbic, *mPFC* medial prefrontal cortex.

^1^ HPRT1 validated among 5 reference genes.

^2^ See Discussion.

### Validation of reference genes in spinal cord dorsal horn over time after SNI

**Figure 1:**
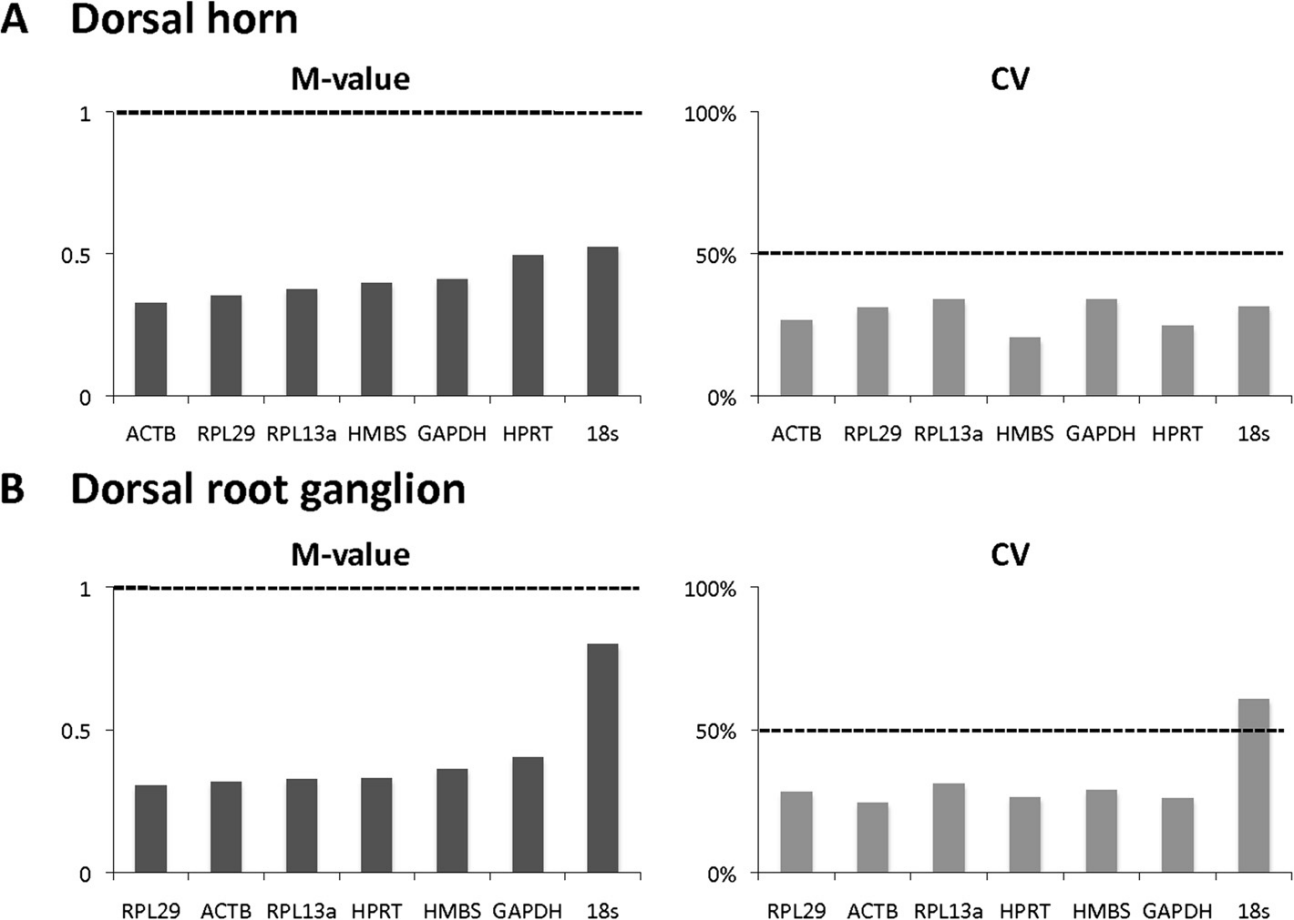
**Analysis of reference gene stability.** Assessment of reference gene stability, in the ipsilateral dorsal horn **(A)** and L4-L5 dorsal root ganglia **(B)** in the rat spared nerve injury model using M-value (left) from the geNorm algorithm and coefficient of variation (CV) (right). Candidate genes are classified from left to right by increasing order of M-value, from most to least stable. The cut-off for stability is 1 for M-value and 50% for CV (dotted line).

Rat spinal cord dorsal horn samples were analyzed in an overall panel without differentiating any timepoints, i.e. throughout a temporal follow-up with naive animals, 2nd, 4th, 7th, 10th, 14th and 21st postoperative day animals. The pairwise comparison of all the potential reference genes (Actb, 18S, GAPDH, RPL13a, RPL29, HPRT1 and HMBS) calculated using geNorm resulted in M-values below 0.5 for all but the 18S gene, which showed 0.52 (Figure 1). All genes were below the M-value cut-off of 1.0 proposed for a heterogeneous panel. CV ranged from 20% for HMBS to 34% for GAPDH and RPL13a, i.e. below the 50% cut-off for stability in a heterogeneous panel.

### Validation of reference genes in DRG after SNI in a time-dependent panel

As for the dorsal horn, DRG samples were analyzed in an overall panel without differentiating any timepoints. M-values were below 1.0 and CV below 50% for Actb, GAPDH, RPL13a, RPL29, HPRT1 and HMBS. The 18S reference gene had the worst M-value, at 0.8, but this was still acceptable within the selection. However, we rejected it from our gene sample because of its 61% CV (Figure 1).

### Ranking of reference genes

We reanalyzed the 7 tested reference genes (Actb, GAPDH, RPL13a, RPL29, HPRT1, HMBS and 18S) to rank them. After each step, the gene with the highest M-value was eliminated until we obtained the two most stable genes from the list. No further discrimination was possible.

**Figure 2:**
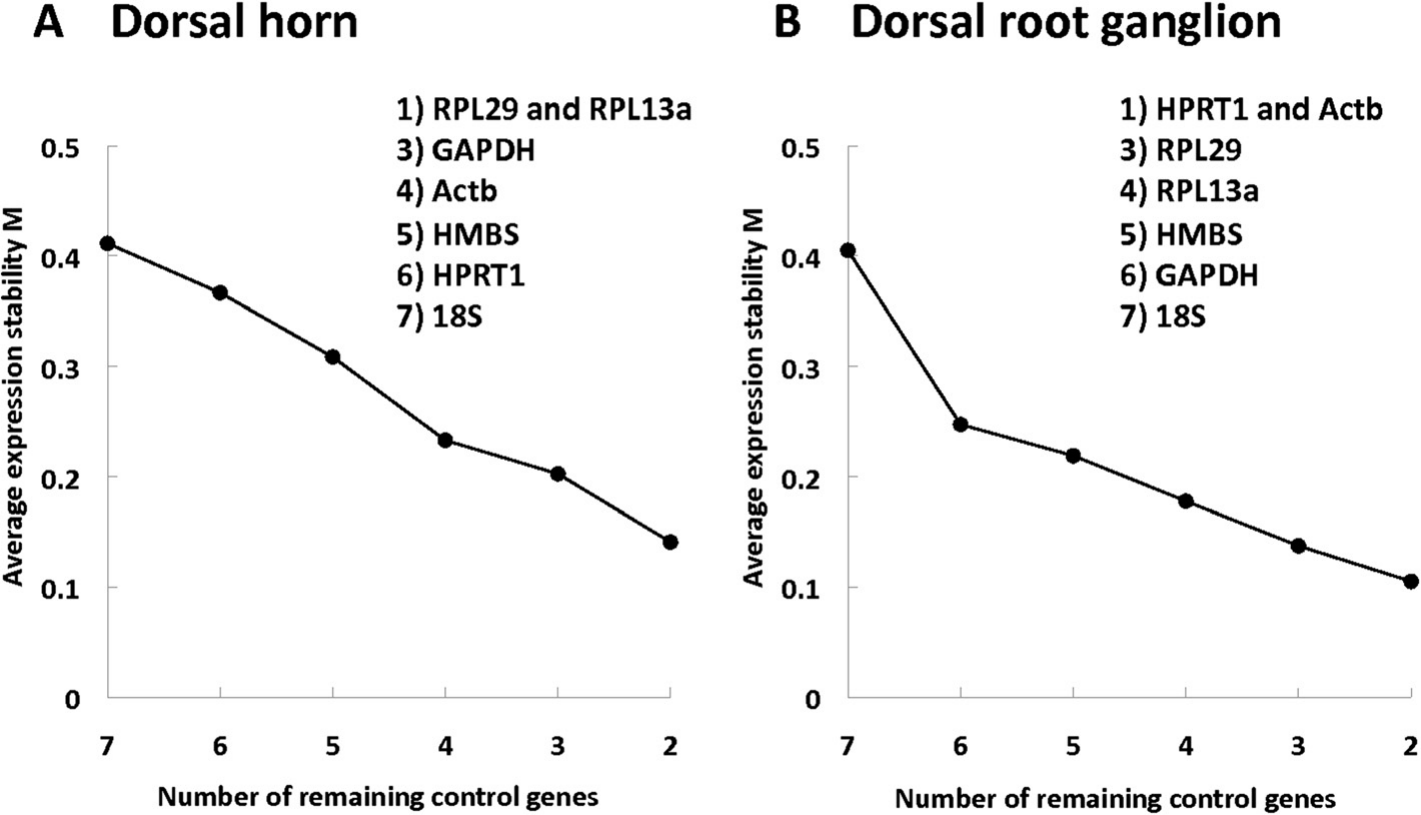
**Ranking of reference genes.** Stepwise calculation of stability by excluding the least stable gene and averaging the remaining M-values in the spinal cord dorsal horn **(A)** and the dorsal root ganglia **(B)**. Insert: list of reference genes ranked from the most to least stable.

In the spinal cord dorsal horn, the 2 most stable genes remaining after the stepwise elimination were RPL29 and RPL13a, and then in increasing order GAPDH, Actb, HMBS, HPRT1 and 18S (Figure 2A).

In the DRG, the 2 most stable genes remaining after the stepwise elimination were HPRT1 and Actb, and then in increasing order RPL29, RPL13a, HMBS, GAPDH and 18S (Figure 2B). The latter is used because this ranking only takes into account the M-value and not the CV.

### Minimal number of reference genes

**Figure 3:**
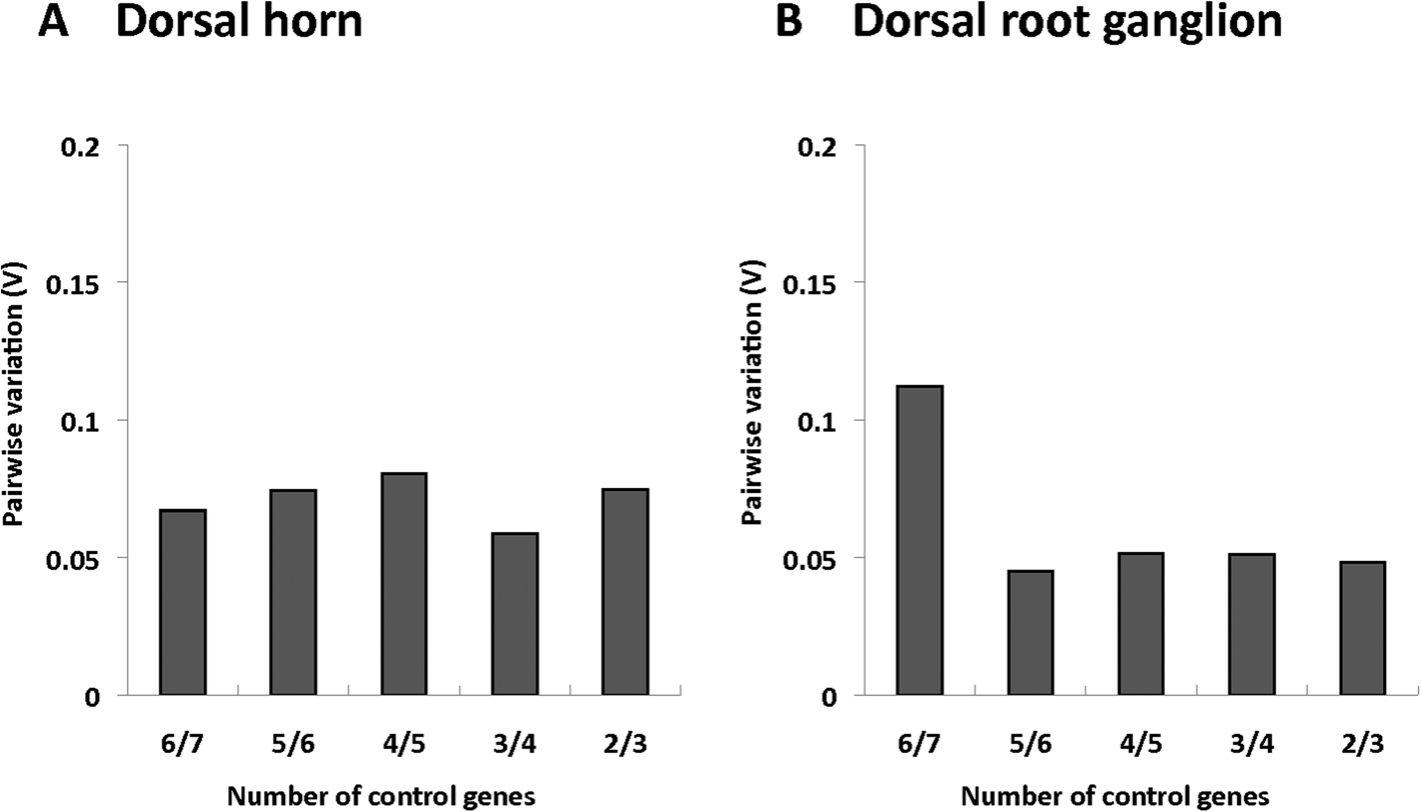
**Determination of the optimal number of control genes for accurate normalization.** Determination of the number of reference genes to consider for an accurate normalization in spinal cord dorsal horn **(A)** and dorsal root ganglia **(B)** by calculating pairwise variation (V) after stepwise elimination of the worst reference gene.

In the dorsal horn samples, the calculation of pairwise variations resulted in values much lower than 0.15 along the entire time course and in all samples. Using the two best reference genes the pairwise variation in dorsal horn was 0.07 (Figure 3). The addition of a supplementary reference gene did not significantly lower the pairwise variation. Results for DRG were similar, with a low pairwise variation of 0.05 if only the two most stable reference genes were taken in consideration.

We performed the same analysis using the least stable reference genes on our list for dorsal horn and DRG, and their pairwise variation was still below 0.15.

## Discussion

Here, we tested the stability of seven candidate reference genes according to the geNorm algorithm to ensure accurate RT-qPCR normalization. M-values below 1.0 together with CV lower than 50% for Actb, GAPDH, RPL13a, RPL29, HPRT1, and HMBS identified these candidate genes as good controls in the rat SNI model in the spinal cord dorsal horn and DRG. The 18S gene could not be validated in DRG because of a CV above 50%.

After SNI, gene expression changes in both sites can be enormous compared to the baseline and also over time [51]. Because of this heterogeneity, meticulous RT-qPCR procedures relying on MIQE recommendations are essential to obtain consistent data. The most frequently chosen reference gene for RT-qPCR normalization after SNI in the rat is GAPDH, but its expression level had, to our knowledge, never been confirmed as being stable in this model (Table 2). Here, we confirm that GAPDH is a good candidate gene, even if it is not the most stable in our series.

The only 2 reference genes with previously mentioned validation in the rat SNI model are HPRT1 and 18S. HPRT1 was used by Shankarappa et al. in DRG [28] at 2 timepoints after injury and chosen from among 5 different reference genes on an array. After showing that 18S had a very low variation of Cq values in all data sets, from both SNI and naive groups, Staaf et al. used it to normalize and compare the expression of the transient receptor potential (TRP) family of genes in DRG after SNI. They mentioned neither ranking between the different reference genes, nor the criteria used to choose 18S from among their list of them [43]. These demonstrations are part of the workflow of RT-qPCR arrays where several reference genes are proposed, and their analysis is usually fully integrated in the procedure.

GeNorm is a popular algorithm for assessing different reference genes from a given panel [9,11]. The calculation of M-values permits measurement of an average variation in the expression ratios of one potential reference gene with the others and consequently informs us whether the tested gene is constantly expressed compared to them. The CV of relative quantities of a gene merely reflects the dispersion of its expression level. It has been previously demonstrated that a CV below 25% was typically observed in stable reference genes as long as the experimental conditions were homogeneous. In a heterogeneous panel, such as in the present study, a CV below 50% is acceptable [11]. As it measures the gene-specific variation, the CV in assessing the stability of a reference gene assumes a standardization of the procedures for all samples. Taken together, M-values and the CV are very useful tools for determining the most accurate reference genes because the information that they convey is different. All our candidate genes satisfied an M-value cut-off below 1.0 for a heterogeneous panel. 18S, despite its acceptable M-value, failed to satisfy the CV cut-off in DRG. Considering these results 18S could not be validated as a stable reference gene.

The more the M-value tends to zero, the more the candidate gene is stable compared to the other tested genes. Hence the stability of the different genes can be ranked. A first approximate classification was established after analyzing all the samples together (Figure 1). Actb, RPL13a and RPL29 were the 3 best genes in both tissues. A potentially more accurate classification can be performed if M-values are recalculated in a series of steps, after elimination of the worst scoring gene at each step. At the end, the two most reliable reference genes remain, but they cannot be further discriminated by definition of the M-value (Figure 2). Interestingly the ranking differed between dorsal horn and DRG. We again found RPL 29 and RPL13a to be the most stable genes in the spinal cord dorsal horn, however, in the DRG, HPRT1 and Actb were the best, followed immediately by RPL13a and RPL29. In the present study, differences in the ranking were very slight, and the former “rough” method would have been sufficient. In other circumstances, or with another panel of candidate genes, it could be useful to compare them further until a definitive ranking is obtained, so as to choose only the best control genes from amongst the candidates. M-value calculations and subsequent ranking were also performed independently for each different time point (data not shown) without being able to identify any time-dependent regulation. The primary goal of this study was to validate some suitable RT-qPCR reference genes at different time points, in different tissues, in a widely used neuropathic pain model.

According to MIQE guidelines, the use of a single reference gene is not enough to compensate for the intrinsic variation within that reference gene and normalization against several reference genes should be performed [7,9]. To define the minimal number of necessary reference genes, the pairwise variation permits an assessment of whether the removal of the worst reference gene may result in a greater variation (Figure 3). Here, in both DRG and dorsal horn, the two best candidate genes were sufficient to ensure an accurate normalization. This demonstration allows us to state that in our model, using only the two best reference genes would be sufficient: namely RPL 29 and RPL13a in the spinal cord dorsal horn and HPRT1 and Actb in the DRG. However, even the worst pair satisfied stability criteria, with a pairwise variation less than 0.15. In fact, all pairs of validated reference genes (not 18S because of its CV above 50%) provided a robust validation in both tissues and at all time points. GAPDH is often used, but according to MIQE guidelines it should not be used alone.

The criterion for choosing a reference gene is more than just its stability. 18S, for example, is disputed as a reference gene by some authors who argue that it by far exceeds the mRNA quantity of most genes of interest and it might be absent from pure mRNA extracts [9].

## Conclusions

We validated 7 reference genes in the spinal cord dorsal horn: (Actin beta (Actb), Glyceraldehyde-3-phosphate dehydrogenase (GAPDH), ribosomal proteins L13a (RPL13a) and L29 (RPL29), hypoxanthine phosphoribosyltransferase 1 (HPRT1), hydroxymethylbilane synthase (HMBS) and 18S. In DRG, 18S did not fulfill all our criteria and could not be retained as a good reference gene, although the other 6 were. We were able to show that in both tissues, using any two of the proposed stable reference genes is sufficient to ensure an accurate validation. We were also able to confirm the stability of GAPDH, which is the most commonly used reference gene in the SNI model, but it should be used in combination with another control gene.

### Availability of supporting data

The data sets supporting this article’s results are available in the Labarchives repository under http://dx.doi.org/10.6070/H4CZ3532.

## Competing interests

The authors declare that they have no competing interests.

## Authors' contributions

NP performed the surgeries, the tissue extraction, the RT-qPCR, the literature search, the statistical analysis and drafted the manuscript. ID participated in the study design and in the drafting of the manuscript. MRS conceived the study, participated in its design and coordination and supervised the drafting of the manuscript. All authors read and approved the final manuscript.

## References

[B1] Bouhassira D Lanteri-Minet M Attal N Laurent B Touboul C Prevalence of chronic pain with neuropathic characteristics in the general population Pain 2008 136 3 380 387 10.1016/j.pain.2007.08.013 17888574

[B2] Breivik H Collett B Ventafridda V Cohen R Gallacher D Survey of chronic pain in europe: prevalence, impact on daily life, and treatment Eur J Pain 2006 10 4 287 333 10.1016/j.ejpain.2005.06.009 16095934

[B3] Decosterd I Woolf CJ Spared nerve injury: an animal model of persistent peripheral neuropathic pain Pain 2000 87 2 149 158 10.1016/S0304-3959(00)00276-1 10924808

[B4] Basbaum AI Bautista DM Scherrer G Julius D Cellular and molecular mechanisms of pain Cell 2009 139 2 267 284 10.1016/j.cell.2009.09.028 19837031 PMC2852643

[B5] Costigan M Befort K Karchewski L Griffin RS D'Urso D Allchorne A Sitarski J Mannion JW Pratt RE Woolf CJ Replicate high-density rat genome oligonucleotide microarrays reveal hundreds of regulated genes in the dorsal root ganglion after peripheral nerve injury BMC Neurosci 2002 3 16 10.1186/1471-2202-3-16 12401135 PMC139981

[B6] Spofford CM Brennan TJ Gene expression in skin, muscle, and dorsal root ganglion after plantar incision in the rat Anesthesiology 2012 117 1 161 172 10.1097/ALN.0b013e31825a2a2b 22617252 PMC3389501

[B7] Bustin SA Benes V Garson JA Hellemans J Huggett J Kubista M Mueller R Nolan T Pfaffl MW Shipley GL Vandesompele J Wittwer CT The MIQE guidelines: minimum information for publication of quantitative real-time PCR experiments Clin Chem 2009 55 4 611 622 10.1373/clinchem.2008.112797 19246619

[B8] Taylor S Wakem M Dijkman G Alsarraj M Nguyen M A practical approach to RT-qPCR-publishing data that conform to the MIQE guidelines Methods 2010 50 4 S1 S5 10.1016/j.ymeth.2010.01.005 20215014

[B9] Vandesompele J De Preter K Pattyn F Poppe B Van Roy N De Paepe A Speleman F Accurate normalization of real-time quantitative RT-PCR data by geometric averaging of multiple internal control genes Genome Biol 2002 3 7 research0034.1-0034.11 10.1186/gb-2002-3-7-research0034 PMC126239 12184808

[B10] Pfaffl MW Tichopad A Prgomet C Neuvians TP Determination of stable housekeeping genes, differentially regulated target genes and sample integrity: bestkeeper–excel-based tool using pair-wise correlations Biotechnol Lett 2004 26 6 509 515 15127793 10.1023/b:bile.0000019559.84305.47

[B11] Hellemans J Mortier G De Paepe A Speleman F Vandesompele J QBase relative quantification framework and software for management and automated analysis of real-time quantitative PCR data Genome Biol 2007 8 2 R19 10.1186/gb-2007-8-2-r19 17291332 PMC1852402

[B12] Gubern C Hurtado O Rodriguez R Morales JR Romera VG Moro MA Lizasoain I Serena J Mallolas J Validation of housekeeping genes for quantitative real-time PCR in in-vivo and in-vitro models of cerebral ischaemia BMC Mol Biol 2009 10 57 10.1186/1471-2199-10-57 19531214 PMC2706836

[B13] Nelissen K Smeets K Mulder M Hendriks JJ Ameloot M Selection of reference genes for gene expression studies in rat oligodendrocytes using quantitative real time PCR J Neurosci Methods 2010 187 1 78 83 10.1016/j.jneumeth.2009.12.018 20036692

[B14] Yao L Chen X Tian Y Lu H Zhang P Shi Q Zhang J Liu Y Selection of housekeeping genes for normalization of RT-PCR in hypoxic neural stem cells of rat in vitro Mol Biol Rep 2012 39 1 569 576 10.1007/s11033-011-0772-8 21633896

[B15] Wan G Yang K Lim Q Zhou L He BP Wong HK Too HP Identification and validation of reference genes for expression studies in a rat model of neuropathic pain Biochem Biophys Res Commun 2010 400 4 575 580 10.1016/j.bbrc.2010.08.106 20804730

[B16] Zimmermann M Ethical guidelines for investigations of experimental pain in conscious animals Pain 1983 16 2 109 110 10.1016/0304-3959(83)90201-4 6877845

[B17] Pertin M Gosselin RD Decosterd I The spared nerve injury model of neuropathic pain Methods Mol Biol 2012 851 205 212 10.1007/978-1-61779-561-9_15 22351093

[B18] Udvardi MK Czechowski T Scheible WR Eleven golden rules of quantitative RT-PCR Plant Cell 2008 20 7 1736 1737 10.1105/tpc.108.061143 18664613 PMC2518243

[B19] Kubista M Andrade JM Bengtsson M Forootan A Jonak J Lind K Sindelka R Sjoback R Sjogreen B Strombom L Ståhlberg A Zoric N The real-time polymerase chain reaction Mol Aspects Med 2006 27 2–3 95 125 16460794 10.1016/j.mam.2005.12.007

[B20] Dieffenbach CW Lowe TM Dveksler GS General concepts for PCR primer design PCR Methods Appl 1993 3 3 S30 S37 10.1101/gr.3.3.S30 8118394

[B21] Ye J Coulouris G Zaretskaya I Cutcutache I Rozen S Madden TL Primer-BLAST: a tool to design target-specific primers for polymerase chain reaction BMC Bioinforma 2012 13 134 10.1186/1471-2105-13-134 PMC3412702 22708584

[B22] Proudnikov D Yuferov V LaForge KS Ho A Jeanne Kreek M Quantification of multiple mRNA levels in rat brain regions using real time optical PCR Brain Res Mol Brain Res 2003 112 1–2 182 185 12670717 10.1016/s0169-328x(03)00056-1

[B23] Tanga FY Raghavendra V DeLeo JA Quantitative real-time RT-PCR assessment of spinal microglial and astrocytic activation markers in a rat model of neuropathic pain Neurochem Int 2004 45 2–3 397 15145554 10.1016/j.neuint.2003.06.002

[B24] Pfaffl MW A new mathematical model for relative quantification in real-time RT-PCR Nucleic Acids Res 2001 29 9 e45 10.1093/nar/29.9.e45 11328886 PMC55695

[B25] Kanda H Kobayashi K Yamanaka H Noguchi K COX-1-dependent prostaglandin D2 in microglia contributes to neuropathic pain via DP2 receptor in spinal neurons Glia 2013 61 6 943 956 10.1002/glia.22487 23505121

[B26] Zhou J Yang CX Zhong JY Wang HB Intrathecal TRESK gene recombinant adenovirus attenuates spared nerve injury-induced neuropathic pain in rats Neuroreport 2013 24 3 131 136 10.1097/WNR.0b013e32835d8431 23370493

[B27] Kashimoto R Yamanaka H Kobayashi K Okubo M Yagi H Mimura O Noguchi K Phosphorylation of ezrin/radixin/moesin (ERM) protein in spinal microglia following peripheral nerve injury and lysophosphatidic acid administration Glia 2013 61 3 338 348 10.1002/glia.22436 23065679

[B28] Shankarappa SA Tsui JH Kim KN Reznor G Dohlman JC Langer R Kohane DS Prolonged nerve blockade delays the onset of neuropathic pain Proc Natl Acad Sci USA 2012 109 43 17555 17560 10.1073/pnas.1214634109 23045676 PMC3491532

[B29] Inquimbert P Bartels K Babaniyi OB Barrett LB Tegeder I Scholz J Peripheral nerve injury produces a sustained shift in the balance between glutamate release and uptake in the dorsal horn of the spinal cord Pain 2012 153 12 2422 2431 10.1016/j.pain.2012.08.011 23021150 PMC3540793

[B30] Zapata A Pontis S Schepers RJ Wang R Oh E Stein A Backman CM Worley P Enguita M Abad MA Trullas R Shippenberg TS Alleviation of neuropathic pain hypersensitivity by inhibiting neuronal pentraxin 1 in the rostral ventromedial medulla J NeuroSci 2012 32 36 12431 12436 10.1523/JNEUROSCI.2730-12.2012 22956834 PMC3457630

[B31] Samad OA Tan AM Cheng X Foster E Dib-Hajj SD Waxman SG Virus-mediated shRNA knockdown of Na(v)1.3 In Rat dorsal root ganglion attenuates nerve injury-induced neuropathic pain Mol Ther 2013 21 1 49 56 10.1038/mt.2012.169 22910296 PMC3538302

[B32] Kobayashi K Yamanaka H Yanamoto F Okubo M Noguchi K Multiple P2Y subtypes in spinal microglia are involved in neuropathic pain after peripheral nerve injury Glia 2012 60 10 1529 1539 10.1002/glia.22373 22736439

[B33] Liu M Zhou L Chen Z Hu C Analgesic effect of iridoid glycosides from paederia scandens (LOUR.) MERRILL (rubiaceae) on spared nerve injury rat model of neuropathic pain Pharmacol Biochem Behav 2012 102 3 465 470 10.1016/j.pbb.2012.06.007 22698486

[B34] Tochiki KK Cunningham J Hunt SP Geranton SM The expression of spinal methyl-CpG-binding protein 2, DNA methyltransferases and histone deacetylases is modulated in persistent pain states Mol Pain 2012 8 14 10.1186/1744-8069-8-14 22369085 PMC3351747

[B35] Okubo M Yamanaka H Kobayashi K Kanda H Dai Y Noguchi K Up-regulation of platelet-activating factor synthases and its receptor in spinal cord contribute to development of neuropathic pain following peripheral nerve injury Mol Pain 2012 8 8 10.1186/1744-8069-8-8 22296727 PMC3293010

[B36] del Rey A Yau HJ Randolf A Centeno MV Wildmann J Martina M Besedovsky HO Apkarian AV Chronic neuropathic pain-like behavior correlates with IL-1beta expression and disrupts cytokine interactions in the hippocampus Pain 2011 152 12 2827 2835 10.1016/j.pain.2011.09.013 22033365 PMC3215892

[B37] Kuhlein HN Tegeder I Moser C Lim HY Haussler A Spieth K Jennes I Marschalek R Beckhaus T Karas M Fauth M Ehnert C Geisslinger G Niederberger E Nerve injury evoked loss of latexin expression in spinal cord neurons contributes to the development of neuropathic pain PLoS One 2011 6 4 e19270 10.1371/journal.pone.0019270 21572518 PMC3084808

[B38] Yamanaka H Kobayashi K Okubo M Fukuoka T Noguchi K Increase of close homolog of cell adhesion molecule L1 in primary afferent by nerve injury and the contribution to neuropathic pain J Comp Neurol 2011 519 8 1597 1615 10.1002/cne.22588 21452236

[B39] de Novellis V Vita D Gatta L Luongo L Bellini G De Chiaro M Marabese I Siniscalco D Boccella S Piscitelli F Di Marzo V Palazzo E Rossi F Maione S The blockade of the transient receptor potential vanilloid type 1 and fatty acid amide hydrolase decreases symptoms and central sequelae in the medial prefrontal cortex of neuropathic rats Mol Pain 2011 7 7 10.1186/1744-8069-7-7 21241462 PMC3031241

[B40] Vega-Avelaira D Geranton SM Fitzgerald M Differential regulation of immune responses and macrophage/neuron interactions in the dorsal root ganglion in young and adult rats following nerve injury Mol Pain 2009 5 70 10.1186/1744-8069-5-70 20003309 PMC2799401

[B41] Costigan M Moss A Latremoliere A Johnston C Verma-Gandhu M Herbert TA Barrett L Brenner GJ Vardeh D Woolf CJ Fitzgerald M T-cell infiltration and signaling in the adult dorsal spinal cord is a major contributor to neuropathic pain-like hypersensitivity J Neurosci 2009 29 46 14415 14422 10.1523/JNEUROSCI.4569-09.2009 19923276 PMC2813708

[B42] Okubo M Yamanaka H Kobayashi K Noguchi K Leukotriene synthases and the receptors induced by peripheral nerve injury in the spinal cord contribute to the generation of neuropathic pain Glia 2010 58 5 599 610 19908283 10.1002/glia.20948

[B43] Staaf S Oerther S Lucas G Mattsson JP Ernfors P Differential regulation of TRP channels in a rat model of neuropathic pain Pain 2009 144 1–2 187 199 19446956 10.1016/j.pain.2009.04.013

[B44] Moss A Ingram R Koch S Theodorou A Low L Baccei M Hathway GJ Costigan M Salton SR Fitzgerald M Origins, actions and dynamic expression patterns of the neuropeptide VGF in rat peripheral and central sensory neurones following peripheral nerve injury Mol Pain 2008 4 62 10.1186/1744-8069-4-62 19077191 PMC2614976

[B45] Berta T Poirot O Pertin M Ji RR Kellenberger S Decosterd I Transcriptional and functional profiles of voltage-gated Na(+) channels in injured and non-injured DRG neurons in the SNI model of neuropathic pain Mol Cell Neurosci 2008 37 2 196 208 10.1016/j.mcn.2007.09.007 17964804

[B46] Millecamps M Centeno MV Berra HH Rudick CN Lavarello S Tkatch T Apkarian AV D-cycloserine reduces neuropathic pain behavior through limbic NMDA-mediated circuitry Pain 2007 132 1–2 108 123 17449176 10.1016/j.pain.2007.03.003 PMC3224847

[B47] Moss A Beggs S Vega-Avelaira D Costigan M Hathway GJ Salter MW Fitzgerald M Spinal microglia and neuropathic pain in young rats Pain 2007 128 3 215 224 10.1016/j.pain.2006.09.018 17110040

[B48] Apkarian AV Lavarello S Randolf A Berra HH Chialvo DR Besedovsky HO del Rey A Expression of IL-1beta in supraspinal brain regions in rats with neuropathic pain Neurosci Lett 2006 407 2 176 181 10.1016/j.neulet.2006.08.034 16973269 PMC1851944

[B49] Pertin M Ji RR Berta T Powell AJ Karchewski L Tate SN Isom LL Woolf CJ Gilliard N Spahn DR Decosterd I Upregulation of the voltage-gated sodium channel beta2 subunit in neuropathic pain models: characterization of expression in injured and non-injured primary sensory neurons J Neurosci 2005 25 47 10970 10980 10.1523/JNEUROSCI.3066-05.2005 16306410 PMC6725885

[B50] Takahashi N Kikuchi S Dai Y Kobayashi K Fukuoka T Noguchi K Expression of auxiliary beta subunits of sodium channels in primary afferent neurons and the effect of nerve injury Neuroscience 2003 121 2 441 450 10.1016/S0306-4522(03)00432-9 14522002

[B51] Scholz J Abele A Marian C Haussler A Herbert TA Woolf CJ Tegeder I Low-dose methotrexate reduces peripheral nerve injury-evoked spinal microglial activation and neuropathic pain behavior in rats Pain 2008 138 1 130 142 10.1016/j.pain.2007.11.019 18215468 PMC2536692

